# Essential Oil Composition of the Different Parts and *In Vitro* Shoot Culture of *Eryngium planum* L

**DOI:** 10.3390/molecules16087115

**Published:** 2011-08-19

**Authors:** Barbara Thiem, Małgorzata Kikowska, Anna Kurowska, Danuta Kalemba

**Affiliations:** 1 Department of Pharmaceutical Botany and Plant Biotechnology, K. Marcinkowski University of Medical Sciences in Poznan, 61-861 Poznan, Poland, Email: kikowska@ump.edu.pl; 2 Institute of General Food Chemistry, Technical University of Lodz, 90-942 Lodz, Poland, Email: danuta.kalemba@p.lodz.pl (D.K.)

**Keywords:** *Eryngium planum*, essential oil composition, falcarinol, *in vitro* shoot culture

## Abstract

The essential oils obtained by hydrodistillation from the different parts (inflorescence, stalk leaves, rosette leaves and root) as well as from *in vitro* shoot culture of *Eryngium planum* L. were analyzed by GC-FID-MS in respect to their chemical composition. The different parts of *E. planum* and *in vitro* shoots showed different yields. The part with higher amount was the inflorescences, followed by the stalk leaves and *in vitro* shoots, rosette leaves and finally roots. The essential oils obtained from rosette leaves and *in vitro*-derived rosettes had totally different composition. Quantitative differences were also found between compounds of intact plant organs. The main components of stalk leaf oil and rosette leaf oil were monoterpene (limonene, α- and β-pinene) and sesquiterpene hydrocarbons. In inflorescence oil *cis*-chrysanthenyl acetate (43.2%) was accompanied by other esters (propionate, butanoate, hexanoate and octanoate) and numerous oxygenated sesquiterpenes. Root oil and *in vitro* shoot oil contained mainly (*Z*)-falcarinol and 2,3,4-trimethylbenzaldehyde. This is the first report on the chemical composition of this species.

## 1. Introduction

The genus *Eryngium* L. (Sea Holly) belonging to the subfamily *Saniculoideae* of the Apiaceae is represented by 317 taxa widespread throughout Central Asia, America, Central and Southeast Europe [1]. Some species, such as *E. foetidum* L., *E. maritimum* L., *E. campestre* L. and *E. creticum* Lam. have been used in traditional medicine worldwide [2]; *E. foetidum* L. (culantro), known as “spiny coriander” is strongly aromatic and contains essential oil valuable for pharmaceutical, perfumery and flavor industries [3]. The pharmacological activities of *Eryngium* species depend mainly on high triterpenoid saponin content [4], but presence of flavonoids [5], namely kaempferol and quercetin glycosides [6], and phenolic acids [7] could play an important role. Coumarin derivatives [8], acetylenes [9,10], as well as rosmarinic acid and chlorogenic acid, known as antioxidants [11], have been described for many *Eryngium* species. Rosmarinic acid accumulation in *in vitro E. planum* cultures was previously investigated by the authors [12]. 

Four of the 26 *Eryngium* species described in Flora Europaea [13] grow in Poland as rare or protected plants. *E. planum* L. (Flat Sea Holly) is a rare herbaceous perennial species of native flora with restricted distribution in Poland [14]. This erect herb has silvery-blue stems of 40–60 cm in height, basal leaves and bluish inflorescences. *E. planum* is used in folk medicine in Europe as Eryngii plani herba and Eryngii plani radix. The presence of the active constituents: phenolic acids, triterpenoid saponins, flavonoids, essential oils and coumarins determines their multidirectional pharmacological activity: diuretic, expectorant, spasmolytic, antitussive, antimycotic, stimulant and appetizer [4,7,15]. The chemistry of *E. planum* has been previously studied, but to our knowledge, no study has dealt with the chemical composition of the essential oil from this species. 

Numerous studies have been carried out using plant *in vitro* cultures as a potential source of valuable constituents. Some difficulties correlated with low or varied concentration of desired compounds in intact plants can be overcome by using plant cell biotechnology. The use of cell and organ cultures, selection of high producing culture and medium optimizations gives the possibility of optimizing the processes of increased secondary metabolites accumulation under controlled conditions. *In vitro* technology offers following benefits: novel products not found in nature, use of rare, endangered or protected plants, independence from climatic factors, elimination of the geographical and political boundaries, shorter and more flexible production cycles and easier fulfillment of GLP and GMP demands [16]. It was shown that numerous bioactive compounds of medicinal value including essential oil components may be accumulated in *in vitro* cultures in higher concentration than in intact plants [17,18].

## 2. Results and Discussion

Some species of Apiaceae have been studied for its high essential oils yield, however Pala-Paul *et al*. [14] reported that the genus *Eryngium* did not contain large amounts of essential oil. The hydrodistillation of the dried aerial parts (inflorescences, leaves and rosette leaves), roots and *in vitro*-derived shoot culture of *E. planum* gave essential oils in yields ranging from 0.05% (roots), 0.07% (rosette leaves), 0.10% (stalk leaves and *in vitro* shoots) to 0.23% (inflorescences). This fact can be explained by the process of essential oil distribution from roots through the plant during the vegetative season [19]. The chemical composition of the oils were analyzed by GC-FID-MS. More than one hundred constituents were identified according to their retention indices (RI) and mass spectra. The identified compounds are presented in Table 1. Similarities were observed in the qualitative composition and some significant differences in the quantitative composition of the oils obtained from stalk leaves and rosette leaves. The main constituents of both oils were monoterpene hydrocarbons (42.0% and 28.4%, respectively) with limonene, α- and β-pinene, β-phellandrene and camphene predominating. The second important group was numerous sesquiterpene hydrocarbons (20.0% and 24.4%, respectively). The main difference was high content of terpinen-4-ol (10.9%) and bornyl acetate (18.1%) in rosette leaf oil. 

The oil obtained from inflorescences of *E. planum* contained mainly *cis*-chrysanthenyl esters: acetate (43.2%), propionate (0.2%), butanoate (3.2%), hexanoate (3.9%), and octanoate (1.9%). The latter two have recently been isolated from this oil and identified by NMR [20]. Numerous oxygenated monoterpenes (51.2%), sesquiterpene hydrocarbons (18%) and oxygenated sesquiterpenes (7.7%) were identified in this oil and the unidentified part of the oil was constituted by compounds of this latter group.

**Table 1:** Chemical composition (%) of essential oil from different organs of intact plants and *in vitro* shoot culture of *Eryngium planum* L.

No.	Compound	RIexp	RIlit	Organ of intact plants				*In vitro* shoot
				L	RL	I	R	
1.	Hexanal	776						0.2
2.	Heptanal	876				0.5		0.1
3.	Santene	882	884		0.2			
4.	Tricyclene	922	927	0.1	0.5			0.1
5.	α-Pinene	932	936	5.4	4.6	11.3	0.1	5.0
6.	Camphene	946	950	0.9	5.4	0.1	0.1	t
7.	1-Octen-3-ol	960	962					0.1
8.	Sabinene	970	973			0.1		0.4
9.	β-Pinene	973	978	9.8	2.1	0.3	0.1	0.3
10.	Octanal	978	981			2.6	0.9	0.8
11.	Myrcene	984	987	0.2	0.1	0.2	0.2	
12.	α-Phellandrene	999	1002	0.2	0.1		t	
13.	3-Carene	1007	1010	3.4	0.5		t	
14.	α-Terpinene	1011	1013	0.4	0.2		t	
15.	p-Cymene	1015	1015	1.4	1.4		0.3	0.1
16.	β-Phellandrene	1023	1023	4.9	1.5			
17.	Limonene	1024	1025	14.7	11.3	0.9	3.2	
18.	γ-Terpinene	1052	1051	0.3	0.3		t	
19.	Nonanal	1077	1081					0.1
20.	p-Cymenene	1080	1075	0.1	t			
21.	Terpinolene	1083	1082	0.2	0.2			
22.	Linalool	1086	1086	t	1.8	0.2	0.1	0.1
23.	Hotrienol	1087	1087			1.4		
24.	Isophorone	1093	1095				0.6	
25.	α-Fenchol	1102	1099	0.2	0.1		0.1	
26.	Camphor	1126	1123	t	0.2			
27.	*trans*-Pinocarveol	1128	1126	0.1	0.2			
28.	*cis*-Verbenol	1134	1132					0.1
29.	*trans*-Verbenol	1138	1136					0.4
30.	Menthone	1144	1142					0.1
31.	*cis*-Chrysanthenol	1150	1147			0.7		
32.	Borneol	1155	1150	0.3	1.1			
33.	Cryptone	1163	1160	0.2	0.1			
34.	**Terpinen-4-ol**	1166	1164	0.6	**10.9**	0.2	0.1	0.1
35.	*p*-Cymen-8-ol	1170	1169	0.1				
36.	α-Terpineol	1177	1176	1.4	2.0			0.1
37.	Safranal	1182	1182					0.1
38.	Fenchyl acetate	1210	1205	0.1	1.7			0.3
39.	Thymol metyl ether	1218	1215	1.2				
40.	*cis*-**Chrysanthenyl acetate**	1248	1253			**43.2**		
41.	**Bornyl acetate**	1274	1270	4.6	**18.1**			
42.	2,3,6-Trimethylbenzaldehyde	1296	1293			1.6	1.0	1.6
43.	α-Terpinyl acetate	1330	1335		0.1		0.1	
44.	**2,3,4-Trimethylbenzaldehyde**	1337	1331			t	**17.4**	**6.2**
45.	Bicycloelemene	1337	1338	0.1	0.5	0.7		
46.	δ-Elemene	1341	1340	0.1	0.6	t		
47.	*cis*-Chrysanthenyl propionate	1342	1342		0.1	0.2	0.6	0.6
48.	α-Cubebene	1354	1355	0.1	0.1	t		
49.	Geranyl acetate	1363	1362		0.1			
50.	α-Ylangene	1378	1376	0.1	0.1		0.1	
51.	α-Copaene	1382	1379	1.6	1.0	0.5	0.1	t
52.	Isoledene	1385	1382	0.3	t	0.1		
53.	β-Bourbonene	1391	1386	1.0	t	0.2	0.2	
54.	β-Elemene	1393	1389	2.1	1.1	1.7	t	
55.	β-Isocomene	1393	1389				0.2	
56.	α-Gurjunene	1418	1413		0.3			
57.	β-Ylangene	1424	1420	0.3	0.7		0.1	
58.	β-Caryophyllene	1427	1424	1.2	1.1	3.2		
59.	*cis*-Chrysanthenyl butanoate	1431				3.2		
60.	γ-Elemene	1432	1429	0.7	1.6	0.8		
61.	β-Copaene	1433	1430	0.7	0.4	0.2		
62.	*trans*-α-Bergamotene	1435	1434				0.4	0.1
63.	(*E*)-β-Farnesene	1444	1446	t	2.0	1.4		23.4
64.	Selina-4(15),6-diene	1446	1450		0.3	t		
65.	α-Humulene	1460	1455	1.1	0.4	0.8		
66.	Aromadendra-1(10),4-diene	1467	1462	0.6		1.1		
67.	(*E*)-β-Ionone	1471	1468	0.1	t			
68.	γ-Muurolene	1478	1474	1.2	0.6	0.2	0.7	
69.	Germacrene D	1485	1486	1.4	8.3	2.3		0.8
70.	β-Selinene	1492	1486	0.9	0.5	0.4		
71.	γ-Amorphene	1496	1492	0.3	0.3	t		
72.	*epi*-Zonarene	1498	1494	0.5				
73.	α-Selinene	1498	1494			0.4		
74.	Bicyclogermacrene	1498	1494		1.6	1.5	0.4	0.4
75.	α-Muurolene	1500	1496	1.4			0.1	
76.	β-Bisabolene	1506	1503	0.4	0.7		0.3	0.3
77.	γ-Cadinene	1511	1507	0.5	0.2	0.3		0.2
78.	β-Sequiphellandrene	1513	1516					0.9
79.	*trans*-Calamenene	1519	1519	0.6	0.3	t	t	t
80.	δ-Cadinene	1522	1520	1.5	1.3	0.8	0.5	
81.	Zonarene	1526	1526	0.5	0.1			
82.	ω-Cadinene	1534	1526	0.1	0.1			
83.	α-Calacorene	1539	1539	0.7	0.2	0.3		
84.	Salviadienol	1550	1549	0.9	0.1	0.5		
85.	Germacrene B	1552	1552			1.1		
86.	Mintoxide	1568	1568	0.8	0.1	t		
87.	Spathulenol	1576	1576	2.2	0.6	0.4	0.1	
88.	Salvial-4(14)-en-1-one	1591	1591	0.9	0.2	0.3		
89.	Carotol	1592	1594	0.1	0.1	0.3		0.1
90.	β-Oplopenone	1599	1598	0.8	0.1			
91.	Torilenol	1606	1607	0.8	0.2	0.5		0.5
92.	1,10-di-*epi*-Cubenol	1623	1623	0.3	0.2	0.1		
93.	*cis*-Chrysanthenyl hexanoate	1630	1628	0.2	0.1	3.9		
94.	β-Acorenol	1633	1633	0.1	0.1	0.2		
95.	T-Muurolol	1637	1637	0.9	0.3	0.2		
96.	β-Eudesmol	1640	1641			0.2		
97.	α-Cadinol	1645	1643	0.5	0.1	0.2		
98.	α-Eudesmol	1655	1653			0.2		
99.	Cadalene	1667	1667	0.9	0.1	0.4		
100.	Eudesma-4(15)-dien-1β-ol	1681	1681	1.3	0.2	0.7		
101.	(*E*)-γ-Atlantone	1689	1691				0.7	0.2
102.	Mintsulphide	1743	1743	0.3	0.6			
103.	6,10,14-Trimethylpentadecan-2-one	1832	1832	2.1	0.1	t		
104.	*cis*-Chrysanthenyl octanoate		1832	1.4		1.9		
105.	Neophytadiene (isomere 2)	1837	1837	0.6	0.5			0.1
106.	Palmitic acid	1955						0.9
107.	(*Z*)-Falcarinol	2011	2005		0.2	0.4	64.4	**49.1**
108.	Linoleic acid							0.4
								
**Total identified**				**86.0**	**93.2**	**94.9**	**93.2**	**94.3**
Aliphatic compounds				0.0	0.0	3.1	0.9	1.3
Monoterpene hydrocarbons				42.0	28.4	12.9	4.0	5.9
Oxygenated monoterpenes				10.3	36.6	51.2	1.6	1.9
Sesquiterpene hydrocarbons				20.0	24.4	18	3.1	26.1
Oxygenated sesquiterpenes				10.6	2.4	7.7	0.8	0.8
**Falcarinol**			****	**0.0** ****	**0.2**	**** **0.4**	**64.4**	**49.1**
Other compounds				3.1	1.2	1.6	18.4	9.7
**Oil yield**			****	**** **0.10**	**0.07**	**** **0.23**	**0.05**	**0.10**

RI_exp_ – Experimental Retention Index, RI_lit_ – Literature Retention Index, L – Stalk Leaves, RL – Rosette Leaves, I – Inflorescence, R – Root, t – trace (percentage value less than 0.05%).

(*Z*)-Falcarinol (64.4%) was found as the major component of root essential oil, followed by 2,3,4-trimethylbenzaldehyde (17.4%) with 2,3,6-trimethylbenzaldehyde (1%). (*Z*)-Falcarinol had been previously identified as one of the main compounds in *Eryngium yuccifolium* Michaux. leaves and stalks [21]. This polyacetylene also dominated in the oil obtained from *in vitro* shoot cultures (49.1%). (*E*)-β-Farnesene (23.4%) and 2,3,4-trimethylbenzaldehyde (6.2%) with 2,3,6-trimethylbenzaldehyde (1.6%) were other important constituents of this oil. The various trimethylbenzaldehyde isomers (2,3,4-trimethylbenzaldehyde, 2,4,5-trimethylbenzaldehyde, 2,3,6-trimethylbenzaldehyde, 2,4,6-trimethylbenzaldehyde) were reported in higher concentration in essential oil in different parts of *E. yuccifolium* Michx. [21], *E. foetidum* L. [3,22,23], *E. corniculatum* Lam. [24], *E. expansum* F. Muell. [25], *E. amethystinum* L. [26], and *E. maritimum* L. [19].

Samples of rosette leaves of intact plants and *in vitro*-derived rosettes (shoot culture) were gathered at the same regeneration phase from different type of soil (ground and *in vitro* culture medium). It was surprising that essential oils obtained from these two populations had totally different composition. Quantitative similarities in oil components were found in two different organs – roots of intact plants and *in vitro* regenerated shoot culture. The major constituents of essential oil in shoot *in vitro* cultures and root were (*Z*)-falcarinol (49.1% and 64.4 % respectively) and 2,3,4-trimethylbenzaldehyde (6.2% and 17.4% respectively). These observations could be explain by vegetation phase of plant, location and type of soil [27,28].

Polyacetylenes such as falcarinol and falcarindiol are wide spread among the Apiaceae plant family [10]. They are common in carrots and related vegetables such as parsley, celery, parsnip and fennel as well as in medicinal plants such as ginseng [29]. They show a wide variety of different pharmacological effects including anti-inflammatory, antiplatelet-aggregatory, cytotoxic and antitumor activity [29,30]. Moreover these aliphatic C_17_-polyacetylenes of the falcarinol-type exhibit anti-bacterial, antifungal and antimycobacterial activities [31]. Falcarinol (heptadeca-1,9-dien-4,6-diyn-3-ol) appears to be the most bioactive compound in the falcarinol-type polyacetylenes group. It has shown a pronounced cytotoxic activity against human tumor cells *in vitro* and it also seems to possess *in vivo* anti-tumor activity [10]. These polyacetylenes have also been shown to be responsible for allergic skin reactions [10]. The beneficial effects of falcarinol-type polyacetylenes occur at nontoxic concentrations and thus represent pharmacologically useful properties indicating that polyacetylenes may be important nutraceuticals. Overall the results suggest that oil from different parts of *in vivo* as well as *in vitro* shoots could be a source of falcarinol and polyacetylenes which are important health promoting compounds. 

## 3. Experimental

### 3.1. Intact Plant

Plants of *E. planum* were collected at the full flowering stage in natural site near Torun, in the Kujawy region of Central Poland. The fruits were gathered from the same place. The voucher specimens from the Department of Pharmaceutical Botany and Plant Biotechnology, K. Marcinkowski University of Medical Sciences in Poznan were deposited in the Herbarium of Institute of Natural Fibres and Medicinal Plant in Poznan. Plants were divided into parts (inflorescence, stalk leaves, rosette leaves and roots) and were air-dried.

### 3.2. In Vitro Shoot Culture

Seedlings of *E. planum* were obtained from the seeds, which were isolated from the ripened fruits after their stratification and scarification. For initiation of *in vitro* cultures, the seeds isolated from fruits were washed with distilled water and dipped in 70% ethanol for 30 s followed by rising in 20% Clorox (5% sodium hypochloride) solution containing two drops of Tween 80 for 5 min. They were finally rinsed three times in sterilized double-distilled water. Shoot tips of axenic seedlings (30-day old) were used for induction of shoot culture and establishment on MS [32] basal medium supplemented with 3% sucrose and plant growth regulators: BAP 1.0 mg·L^−1^, and IAA 0.1 mg·L^−1^. Media were solidified with 0.8% agar and adjusted to pH 5.7-5.8, autoclaved at 121 °C for 20 min (105 kPa). Shoot cultures were maintained in 250 cm^3^ Erlenmeyer flasks with 50 cm^3^ of culture medium, subcultured to fresh medium every 6–8 weeks and incubated in growth chamber under a 16/8 h photoperiod at 55 μmol m^−2^ s^−1^ light provided by cool-white fluorescent lamps and a temperature of 23 ± 2 °C. The ‘shoot culture’, a type of *in vitro* cultures, in case of *E. planum* is a rosette of leaves (like a juvenile stadium of intact plant). For isolation of essential oil the multiplied *in vitro* shoots were washed from medium and air dried. 

### 3.3. Isolation and Analysis of Essential Oil

The essential oils were obtained by hydrodistillation for three hours of dried plant material using a glass Clevenger-type apparatus, according to European Pharmacopoeia 5.0. GC-FID-MS analyses were performed using a Trace GC Ultra apparatus (Thermo Electron Corporation) equipped with FID and MS DSQ II detectors and FID-MS splitter (SGE). Operating conditions: apolar capillary column Rtx-1ms (Restek), 60 m × 0.25 mm i.d., film thickness 0.25 µm; temperature program, 50–300 °C at 4 °C/min; SSL injector temperature 280 °C; FID temperature 300 °C; split ratio 1:20; carrier gas helium at a regular pressure 200 kPa. Mass spectra were acquired over the mass range 30–400 Da, ionization voltage 70 eV; ion source temperature 200 °C.

Identification of components was based on the comparison of their MS spectra with those of a laboratory-made MS library, commercial libraries (NIST 98.1, Wiley Registry of Mass Spectral Data, 8th Ed. and MassFinder 4.1, laboratory-made list) and with literature data [33,34] along with the retention indices (Rtx-1, MassFinder 4.1) associated with a series of alkanes with linear interpolation (C_8_-C_26_). A quantitative analysis (expressed as percentages of each component) was carried out by peak area normalization measurements without correction factors.

## 4. Conclusions

The results suggest that oil from different parts of *in vivo E. planum* plants as well as *in vitro* shoots could be a source of falcarinol, polyacetylene which is an important health promoting compound. Our studies have shown that the yield of the oil isolated from different parts of *E. planum* and *in vitro* shoots is low and the essential oils contain complex mixtures of up to 111 different compounds. 
